# Microparticles Induce Cell Cycle Arrest Through Redox-Sensitive Processes in Endothelial Cells: Implications in Vascular Senescence

**DOI:** 10.1161/JAHA.112.001842

**Published:** 2012-06-22

**Authors:** Dylan Burger, Dylan G. Kwart, Augusto C. Montezano, Naomi C. Read, Christopher R.J. Kennedy, Charlie S. Thompson, Rhian M. Touyz

**Affiliations:** Kidney Research Centre, Ottawa Hospital Research Institute, University of Ottawa, Ottawa, Ontario, Canada (D.B., D.G.K., A.C.M., N.C.R., C.R.J.K., R.M.T.); Neuroscience Program, Ottawa Hospital Research Institute, University of Ottawa, Ottawa, Ontario, Canada (C.S.T.); Department of Cellular and Molecular Medicine, University of Ottawa, Ottawa, Ontario, Canada (N.C.R., C.R.J.K.); BHF Glasgow Cardiovascular Research Centre, University of Glasgow, Glasgow, United Kingdom (R.M.T.)

**Keywords:** cell cycle, endothelium, microparticles, oxidative stress, senescence

## Abstract

**Background:**

Chronic disease accelerates endothelial dysfunction in aging, a process associated with cell senescence. However, the mechanisms underlying this process are unclear. We examined whether endothelial cell (EC)-derived microparticles (MPs) facilitate EC senescence and questioned the role of reactive oxygen species in this process.

**Methods and Results:**

Senescence was induced by sequential passaging of primary mouse ECs. Cells retained phenotypic characteristics of ECs from passage 4 through passage 21. Passage 21 ECs exhibited features of senescence, including increased staining of senescence-associated β-galactosidase (SA-βgal), a greater percentage of cells in G_1_/G_0_ phase of the cell cycle, and increased phosphorylation of p66^Shc^ (*P*<0.05). Microparticle formation from passage 21 ECs was increased versus passage 4 ECs (∼2.2-fold increase versus passage 4, *P*<0.05), and the Rho kinase inhibitor fasudil blocked this increase. Exposure of passage 4 ECs to MPs shifted cells from a proliferating to a nonproliferating phenotype, as indicated by cell cycle analysis and increased senescence-associated β-galactosidase staining. MPs increased EC generation of O_2_^•−^ (∼2.7-fold) and H_2_O_2_ (∼2.6-fold), effects blocked by apocynin (nicotinamide adenine dinucleotide phosphate oxidase inhibitor) and rotenone (mitochondrial oxidase inhibitor) but not by allopurinol (xanthine oxidase inhibitor). MPs increased expression of cell cycle proteins p 21 cip1 and p16ink4a and stimulated phosphorylation of p66^Shc^ in ECs (*P*<0.05 versus untreated ECs). Pretreatment with the reactive oxygen species scavenger sodium 4,5-dihydroxybenzene-1,3-disulfonate (tiron) abrogated the prosenescent effects of MPs.

**Conclusions:**

MPs promote EC senescence through nicotinamide adenine dinucleotide phosphate oxidase- and mitochondrial-derived reactive oxygen species. Such redox-sensitive processes may be important in vascular dysfunction in aging. **(*J Am Heart Assoc*. 2012;1:e001842 doi: 10.1161/JAHA.112.001842.)**

## Introduction

Aging is a primary risk factor for the development of cardiovascular disease and is associated with impaired vascular function.^1^ With aging, structural and functional changes in the vasculature occur gradually and include increased arterial thickness, reduced lumen diameter, and a shift to a dysfunctional endothelium, with impaired vasorelaxation, a pro-oxidative, proinflammatory phenotype, and decreased antithrombotic capacity.^2,3^ In chronic disease states, such as hypertension, these age-associated changes are accelerated in a process termed “early vascular aging.”^4,5^ Evidence indicates that during both normal and early vascular aging, healthy endothelial cells (ECs) progressively enter into a senescent, nonreplicative state in which they are metabolically active but do not respond to mitogenic stimuli.^4,5^ Many of the alterations in senescent ECs are consistent with the decline in endothelial function during vascular aging. In this regard, EC senescence is associated with increased reactive oxygen species (ROS) production, decreased nitric oxide production, increased expression of cellular adhesion molecules, increased adhesion of monocytes, and increased sensitivity to proapoptotic stimuli. ^6^ Accordingly, EC senescence has been implicated in the pathogenesis of endothelial dysfunction in early and normal vascular aging.^7^

A central component of vascular aging is the increased production of ROS.^8,9^ In the endothelium, sources of aging-related increases in ROS production include mitochondria, uncoupled endothelial nitric oxide synthase (eNOS), and nicotinamide adenine dinucleotide phosphate (NADPH) oxidase.^9,10^ Regardless of the source, increased ROS has been implicated in the functional alterations of the endothelium as well as in EC inflammation, apoptosis, and senescence.^8,10^ These impairments appear causally linked to longevity, as mice that lack the gene encoding p66^Shc^ exhibit reduced systemic and intracellular ROS, improved endothelial function, and a 30% increase in lifespan.^11,12^ However, while ROS-mediated effects on EC senescence and vascular dysfunction are well established, the stimuli responsible for ROS-mediated increases in EC senescence are unclear.

Associated with EC damage and endothelial dysfunction is the formation of endothelial microparticles (MPs).^13–15^ MPs are small heterogeneous membranous structures (0.1–1 μm), formed from activated or stressed cells as a result of cytoskeletal reorganization, membrane blebbing, and shedding of membrane fragments into extracellular space.^13^ MPs of endothelial, platelet, and leukocyte origin are consistently detectable in plasma samples, are increased under conditions of vascular stress/dysfunction, and appear to reflect vascular health.^13,15^ Recently, our laboratory and others have reported that MPs exert direct effects on the endothelium by increasing endothelial oxidative stress and inflammation, decreasing NO production, or stimulating platelet and macrophage adhesion to ECs.^16–18^ However, the impact of aging on MP formation and effects of MPs on EC senescence are unclear.

In the present study, we tested the hypothesis that MP formation is increased in senescent ECs and that MPs promote premature endothelial senescence through redox-sensitive processes.

## Methods

### Cell Culture

The study was approved by the Animal Ethics Committee of the University of Ottawa and performed according to the recommendations of the Canadian Council for Animal Care. ECs were isolated from the aortas of C57BL6 mice and cultured as described previously.^17^ ECs were seeded on attachment factor-coated polystyrene dishes in Dulbecco's modified Eagle's medium (DMEM, Gibco) containing 10% FCS (Gibco), 50 mg/L of EC growth supplement (Sigma), 10 U/mL heparin, 100 U/mL penicillin/streptomycin, and 1× minimal essential amino acids (Gibco) and placed in a humidified incubator at 37°C and 5% CO_2_.

For studies involving replicative senescence, ECs were cultured in 10% FCS as mentioned earlier. When cells reached approximately 80% confluence, serial passage was performed (1:5 split ratio) by trypsinization with trypsin-EDTA (Gibco). Experiments used cells in either passage 4 (p4; young, little senescence) or passage 21 (p21; old, highly senescent). Cell viability was assessed by Trypan blue dye exclusion.

For studies examining stress-induced premature senescence, ECs were cultured in 10% FCS as above, and cells were stimulated with either 100 μmol/L H_2_O_2_ or MPs (10^5^/mL) in the presence and absence of the O_2_^•−^ scavenger (O_2_^•−^ semiquinone-forming) sodium 4,5-dihydroxybenzene-1,3-disulfonate (tiron; 10 μmol/L, Sigma-Aldrich). Tiron forms a semiquinone radical through an electron trapping transfer from O_2_^•−^. The doses of H_2_O_2_ and MPs were chosen from pilot experiments aimed at identifying optimal induction of senescence. All experiments in which premature senescence was induced were performed in cells from p4 or p5.

### Immunocytochemistry

ECs were seeded on glass slides, cultured to subconfluence, and fixed in 4% paraformaldehyde for 15 minutes. Cells were then incubated overnight at 4°C with a fluorescein isothiocyanate–conjugated antibody to vascular endothelial cadherin (Santa Cruz Biotechnology, 1:100) or unconjugated rabbit anti-eNOS (Cell Signaling, 1:100). For eNOS labeling, protein was visualized by incubation with a fluorescein isothiocyanate–conjugated secondary antibody (1:500, BD Biosciences) for 1 hour. Nuclei were stained with Hoechst 33342 (1 μg/mL), and slides were mounted with fluorescent mounting medium (Dako Scientific) and visualized with a Zeiss Axioskop 2 MOT.

### EC MP Isolation, Quantification, and Electron Microscopy

Endothelial MPs were isolated from media collected from EC cultures as previously described.^17^ Briefly, samples were centrifuged at 1500*g* for 20 minutes at 20°C to obtain cell-free media. MPs were then pelleted from cell-free media by centrifugation at 18 000*g* for 20 minutes at 20°C. Endothelial MPs were confirmed as such and quantified by flow cytometry with an Alexa-647–labeled Annexin V (0.5 μg/mL, Biolegend) to identify events as MPs. As a negative control, a subpopulation of MPs was resuspended in Annexin V binding buffer lacking calcium, which is necessary for Annexin V binding to phosphatidylserine. MPs were centrifuged and resuspended in PBS for treatment. A final concentration of 10^5^ endothelial MPs/mL, previously identified to exert effects on cultured ECs, was used for all experiments.^17^

In addition, MPs from low- and high-passage ECs were examined by electron microscopy. MP-containing suspensions were centrifuged at 18 000*g* for 20 minutes at 20°C, the supernatants were aspirated, and pellets were fixed in 2.5% gluteraldehyde in 1× PBS (overnight at 20°C). The pellets were then washed in 0.1 mol/L Na cacodylate buffer, postfixed in 2% OsO4, and dehydrated in graded ethanol. Samples were embedded in Spurrs Resin, and 60-nm sections were prepared on copper grids. Samples were visualized on a Hitachi H7100 electron microscope. MPs were identified as small (0.1–1.0 μm), rounded objects with clear, intact membranes, as described previously.^17^

### Cell Cycle Analysis

Cell cycle analysis was conducted by using a modified propidium iodine–based flow cytometry protocol.^20,21^ Subconfluent cells were trypsinized and centrifugated at 1000*g* for 5 minutes. The supernatant was discarded and pelleted cells washed in PBS and centrifuged as before. Cells were resuspended in 300 μL PBS and fixed in 70% ethanol at 4°C overnight. Fixed cells were pelleted at 8000*g* for 10 minutes and then incubated in KRISHIAN buffer (0.1% sodium citrate, 0.02 mg/mL RNAse A [Sigma-Aldrich], 0.3% NP-40 [Sigma-Aldrich], and 0.05 mg/mL propidium iodide [Invitrogen]) for 1 hour at 4°C in the dark. Cell suspensions were filtered and analyzed for propidium iodide labeling of DNA by flow cytometry.

### Measurement of Rho Kinase Activity

Rho kinase (ROCK) activity was assessed in ECs with the ROCK Activity Assay Kit (Cell Biolabs Inc) as described previously.^17^

### Measurement of Superoxide and Hydrogen Peroxide Production

Superoxide (O_2_^•−^) and hydrogen peroxide (H_2_O_2_) production was measured in ECs by dihydroethidium high-performance liquid chromatography (HPLC) and the Amplex Red hydrogen Peroxide/Peroxidase Assay Kit (Molecular Probes), respectively, as we have previously described.^17,22^

### Western Blot Analysis

Western blotting was used to examine levels of cell cycle/senescence and autophagy-related proteins in ECs and to determine protein levels in endothelial MPs. Membranes were probed with anti-phospho (ser36) p66-^Shc^ (1:1000; Calbiochem), anti–total-Shc (1:1000, Calbiochem), anti–p16- ink4a (1:500, Santa Cruz Biotechnology), anti-p21cip1 (1:2000; Santa Cruz Biotechnology), anti-p27kip1 (1:2000; Santa Cruz Biotechnology), anti-Bax (1:1000; Santa Cruz Biotechnology), anti-Bcl-2 (1:1000; Santa Cruz Biotechnology), anti–microtubule-associated protein 1 Light Chain 3 (LC3, 1:1000, Cell Signaling Technology), anti-eNOS (1:1000, Cell Signaling Technology), anti–vascular endothelial cadherin (1:1000, Santa Cruz Biotechnology), anti–flotillin-2 (1:4000, BD Biosciences), anti–caveolin-1 (1:2000, Santa Cruz Biotechnology), and anti-GAPDH (1:4000, Millipore). Membranes were then washed in Tris buffered saline with tween-20 and incubated with horse radish peroxidase–conjugated secondary antibodies (1:2000; Santa Cruz Biotechnology) in milk for 1 hour. Membranes were probed for immunoreactivity by chemiluminescence. Quantification of blots was performed by densitometry (ImageJ).

### β-Galactosidase Cytochemical Staining Assay

Senescence-associated β-galactosidase (SA-βgal) activity at pH 6 was measured with a SA-βgal Staining Kit (Cell Signaling Technology). SA-βgal activity at pH 6 is a well-characterized hallmark of senescence, absent in replicating, quiescent, and immortal cells. ^23^ Cultured ECs in 35-mm 6-well plates were washed in 1× PBS, and. A total of 1 mL of Fixative Solution (2% formaldehyde, 0.2% glutaraldehyde, 1× PBS) was added to each well for 15 minutes. Plates were washed in PBS, and then β-galactosidase Staining Solution (40 mmol/L citric acid/sodium phosphate pH 6, 0.15 mol/L NaCl, 2 mmol/L MgCl_2_, 500 nmol/L potassium ferrocyanide, 50 μL 20 mg/mL X-gal in N-N-dimethylformamide) was added to each well and incubated overnight at 37°C. Cells were overlayed with 70% glycerol and stored at 4°C. Slides were imaged via Axiovision 4.6 software with an Axio HRC camera (Zeiss). Twenty-five high-powered fields imaged at 200× magnification (approximately 1000 cells) were analyzed.

### Fluorescence-Based Caspase-3 Activity Assay

Caspase-3 activity was measured with the QuantiZyme Assay System (BioMol) as described previously.^24^ Briefly, cells were lysed in a buffer containing 1 mol/L HEPES (pH 7.4), 0.2% Triton X-100, 140 μg/mL dithiothreitol, and 0.5 mol/L EDTA. Cell lysates (50 μg protein/sample) were then incubated at 37°C for 40 hours in the presence of the BioMol AC-DEVD-AMC Substrate (200 μmol/L) with and without the BioMol AC-DEVD-CHO inhibitor (4 μmol/L) in a buffer containing 1 mol/L HEPES (pH 7.4), 3 mol/L NaCl, 140 μg/mL dithiothreitol, 0.5 mol/L EDTA, 100% glycerol, and 0.2% Triton X-100. Fluorescence was measured on a FLUOstar Galaxy microplate reader (Inter Departmental Equipment) with an excitation wavelength of 360 nm and an emission wavelength of 460 nm. Results are expressed as arbitrary units (AU)/μg protein.

### Statistical Analysis

Results are expressed as mean ± SEM and were analyzed by using a Mann-Whitney test and one-way or two-way ANOVA with a Bonferoni's posttest as appropriate. For parametric statistical tests, data were verified to not deviate from Gaussian distributions with a Kolmogorov**-**Smirnov test. Bonferoni's correction was applied when *P* values were calculated for multiple comparisons. All statistical analyses were performed with Graphpad Prism 4.0 (GraphPad Software, Inc). *P*<0.05 was considered significant.

## Results

### In Vitro Model of Aged ECs

**Figure 1.**
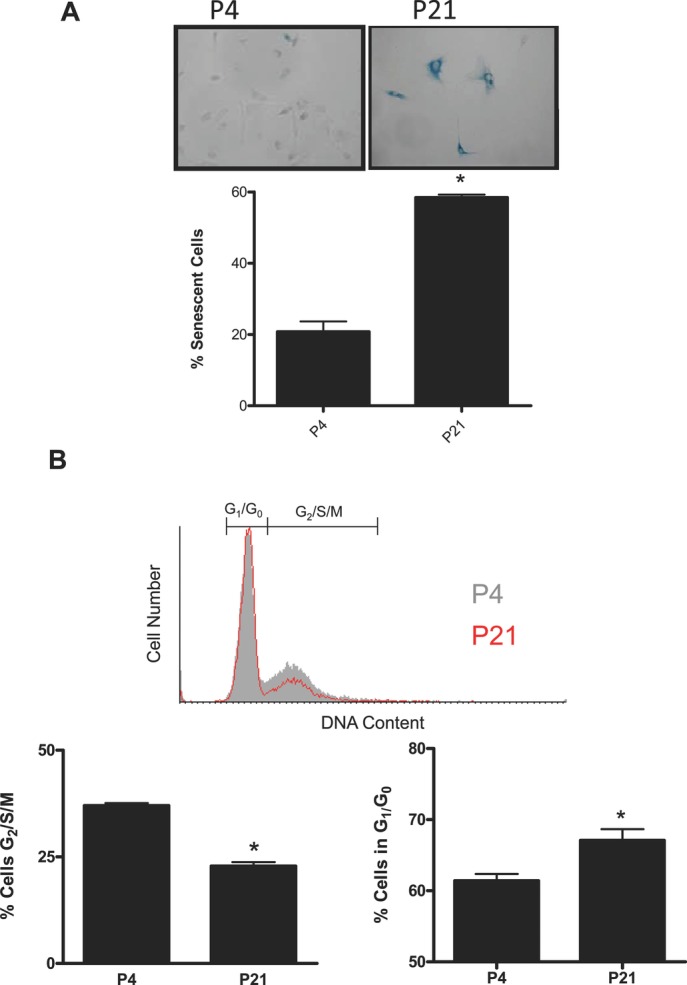
SA-βgal activity and cell cycle analysis in aged ECs. Mouse aortic ECs were serially passaged from p4 to p21. A, SA-βgal staining in p4 and p21 ECs expressed as a percentage of the total EC population. Inset: representative image. B, Distribution of cells in G_1_ and G_2_/S/M expressed as a percentage of total cells. Inset: representative histograms of DNA content during cell cycle; p4 populations are visible as a shaded gray histogram, while p21 populations are visible as the red open histogram. Data are expressed as mean±SEM; *P<0.05 vs p4 cells, n=4.

**Figure 2.**
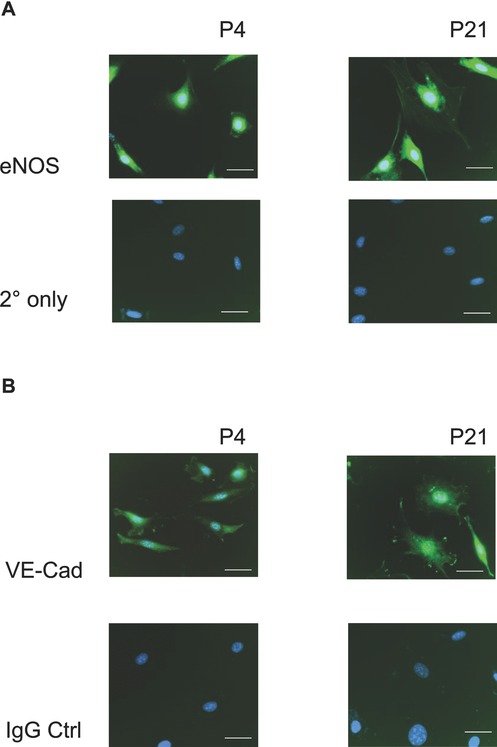
Fluorescent micrographs of p4 and p21 mouse aortic ECs. A, eNOS expression in p4 and p21 ECs. Green fluorescence (FITC) is eNOS; blue fluorescence (4′,6-diamidino-2-phenylindole, DAPI) is Hoechst 33342 nuclear stain. Shown are representative micrographs as well as autofluorescence controls consisting of cells exposed to secondary antibody only. B, VE-cadherin expression in p4 and p21 ECs. Green fluorescence (FITC) is VE-cadherin; blue fluorescence (DAPI) is Hoechst 33342 nuclear stain. Shown are representative micrographs as well as autofluorescence controls consisting of cells exposed to a FITC-conjugated isotype control. Bar represents 10 μm. FITC indicates fluorescein isothiocyanate; VE-cadherin, vascular endothelial cadherin.

**Figure 3.**
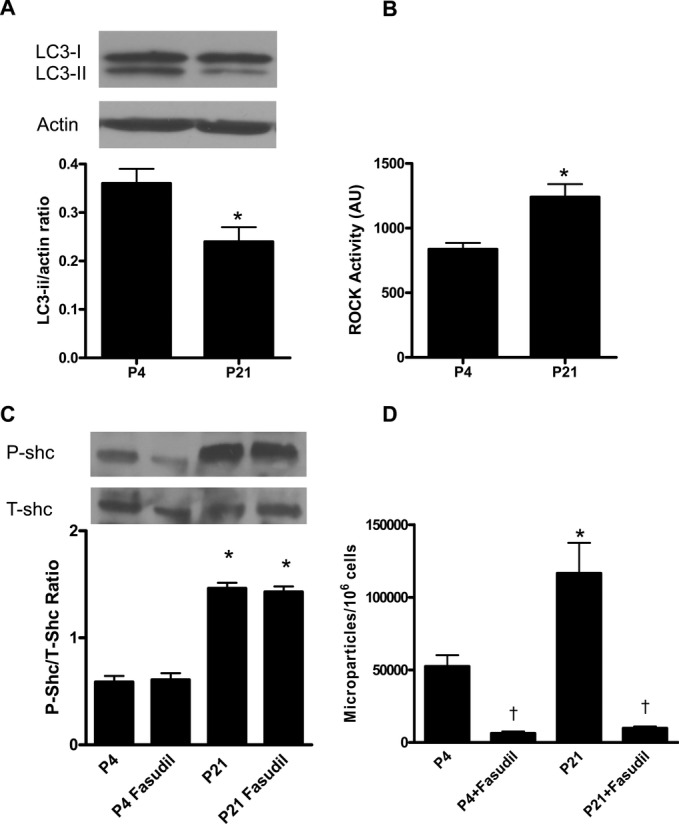
Characterization of p4 and p21 ECs. A, Western analysis of LC3-I and LC3-II levels in p4 and p21 ECs. Levels of LC3-II, the active form, were significantly reduced in p21 cells. Inset: representative blot. Data are expressed as mean±SEM; *P<0.05 vs p4, n=4. B, ROCK activity was significantly increased in p21 vs p4 ECs. Data are expressed as mean±SEM; *P<0.05 vs p4, n=4. C, Phosphorylation of p66^Shc^ was increased in p21 cells compared with p4 cells. Coculture with fasudil (10 μmol/L, 24 hours) had no effect on p66^Shc^ phosphorylation. Data are expressed as mean±SEM; *P<0.05 vs p4, †*P*<0.05 vs untreated cells, n=6. D, MP formation from p4 and p21 ECs was determined by measuring the amount of Annexin V^+ve^ MPs released into media over 24 hours and expressed as the number of MPs/10^6^ cells. Data are expressed as mean±SEM; *P<0.05 vs p4, †*P*<0.05 vs untreated cells, n=6 to 7.

**Figure 4.**
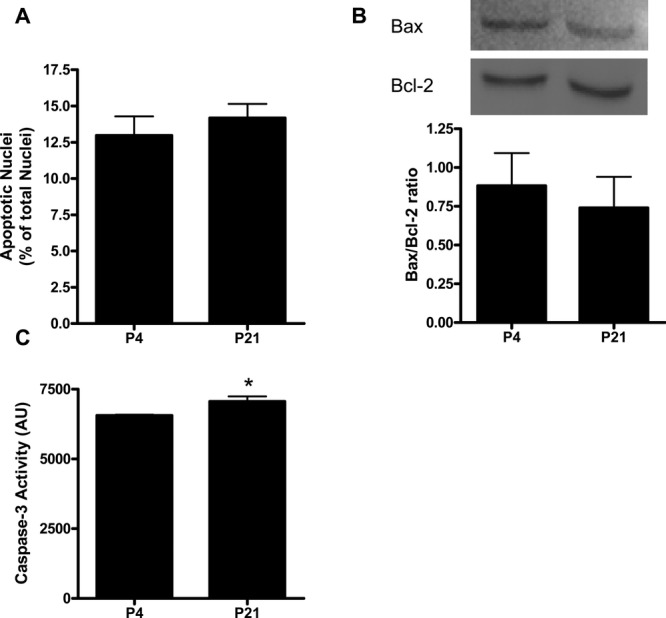
Assessment of apoptosis in p4 and p21 ECs. Mouse aortic ECs were serially passaged from p4 to p21, and apoptosis was assessed by Hoechst nuclear staining (A), Bax/Bcl-2 ratio (B), and caspase-3 activity (C). Inset: representative blot. Data are expressed as mean±SEM; *P<0.05 vs p4 cells, n=5 to 6.

An in vitro model of replicative senescence was used with cultured mouse aortic ECs. Long-term culture of ECs was used as a model of endothelial aging and is associated with decreased proliferation and increased SA-βgal activity.^25,26^ In our studies, culture of mouse aortic ECs to p21 was associated with an increase in the number of cells staining positive for SA-βgal activity (Figure 1A) and a reduction in the number of proliferating cells as determined by propidium iodide cell cycle analysis (Figure 1B). Senescent cells appeared larger and more granular with occasional vacuolization of the cytoplasm (Figure 1A). Importantly, cells retained expression of EC markers including eNOS and vascular endothelial cadherin (Figure 2). Both short-term (p4) and long-term (p21) cultured ECs were viable as assessed by Trypan Blue exclusion (96±1% and 95±2%, respectively, n=5–6). In addition to displaying a senescent phenotype, p21 ECs were characterized by a reduction in levels of LC3-II (Figure 3A), which is indicative of reductions in autophagy,^27^ increased ROCK activity (Figure 3B), and increased phosphorylation of the longevity determinant adaptor protein p66^Shc^ (Figure 3C). No differences in the percentage of apoptotic nuclei (Hoechst staining) or Bax/Bcl-2 ratio were observed (Figure 4A and 4B) suggesting that levels of apoptosis were similar between early- and late-passage ECs. A small but significant increase in levels of caspase-3 activity was observed in late-passage ECs (Figure 4C).

### MP Formation Is Increased in Aged ECs

To examine the formation of MPs in aged ECs, media was collected from p4 and p21 ECs over 24 hours, and the number of Annexin V^+ve^ MPs released into the media was quantified by flow cytometry. Long-term culture of ECs was associated with a significant increase in the rate of spontaneous MP formation from ECs (Figure 3D). On the basis of the observation that ROCK activity was increased in late-passage ECs, we examined whether ROCK plays a mechanistic role in MP formation in senescent ECs. Inhibition of ROCK with fasudil (10 μmol/L) significantly reduced MP formation in both p4 and p21 cells (Figure 3D). Conversely, inhibition of ROCK had no effect on phosphorylation of p66^Shc^ (Figure 3C), suggesting that p66^Shc^ does not play a downstream role in regulating MP formation.

**Figure 5.**
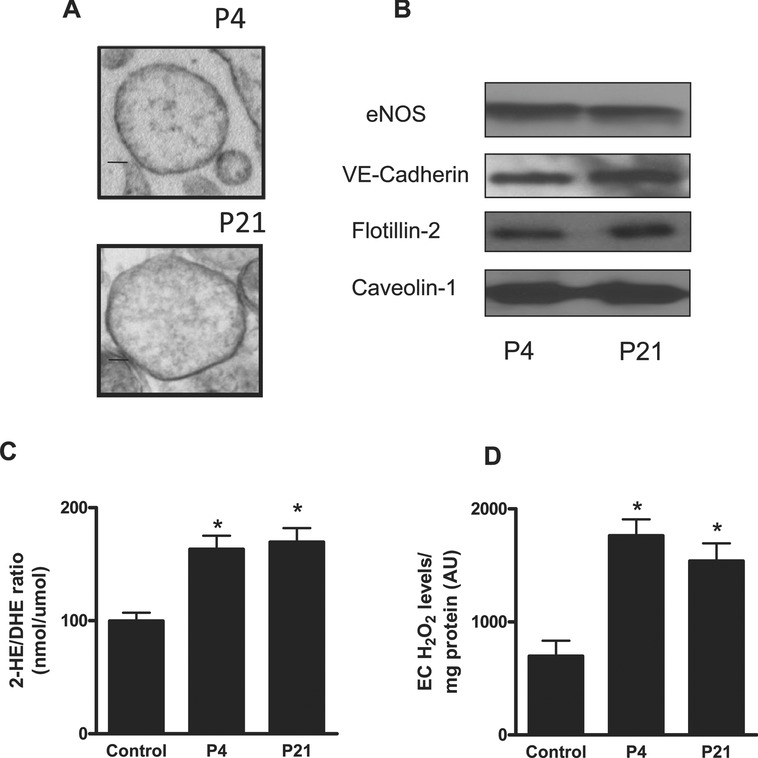
Characterization of EC-derived MPs from p4 and p21 ECs. A, Transmission electron micrographs of MPs from p4 and p21 ECs. MPs were isolated from media, and samples were fixed and examined by electron microscopy at 50 000× magnification. Bar=0.1 μm. B, MPs were isolated from the medium of p4 and p21 ECs, and expression of eNOS, VE-cadherin, flotillin-2, and caveolin-1 was measured by Western blot analysis. C and D, Pro-oxidative effects of endothelial MPs. ECs were cultured and treated with endothelial MPs (10^5^ MPs/mL) for 4 hours, and superoxide (O_2_^•−^) production was measured by DHE/HPLC (C) and H_2_O_2_ was measured by amplex red (D). Treatment with both p4 and p21 MPs was associated with significant increases in O_2_^•−^ and H_2_O_2_ production in ECs. Data are expressed as mean±SEM; *P<0.05 vs control untreated cells, n=5 to 7. FITC indicates fluorescein isothiocyanate.

Characterization of MPs by electron microscopy demonstrated no obvious phenotypic difference between MPs derived from senescent (p21) ECs and MPs from young (p4) ECs (Figure 5A). Similarly, the levels of EC markers vascular endothelial cadherin and eNOS were comparable between p4- and p21-derived MPs (Figure 5B). Levels of caveolin-1 and flotillin-2, markers of lipid rafts/caveolae, which we previously identified as highly expressed in endothelial MPs, were comparable in p4 and p21 cells (Figure 5B).

### MPs Derived From Senescent and Young ECs Promote Oxidative Stress

Our laboratory and others have reported that MPs promote oxidative stress in cultured ECs.^16,17^ To determine whether functional differences exist between MPs formed from healthy and senescent ECs, low-passage ECs were treated with MPs derived from young (p4) and aged (p21) ECs. Treatment of ECs with MPs was associated with a significant increase in O_2_^•−^ (Figure 5C) and H_2_O_2_ (Figure 5D) formation as measured by dihydroethidium (DHE)-HPLC and Amplex Red, respectively. However, no differences were observed between ECs treated with p4 and p21 MPs (Figure 5C and 5D).

### MPs Induce Premature Senescence in Cultured ECs

**Figure 6.**
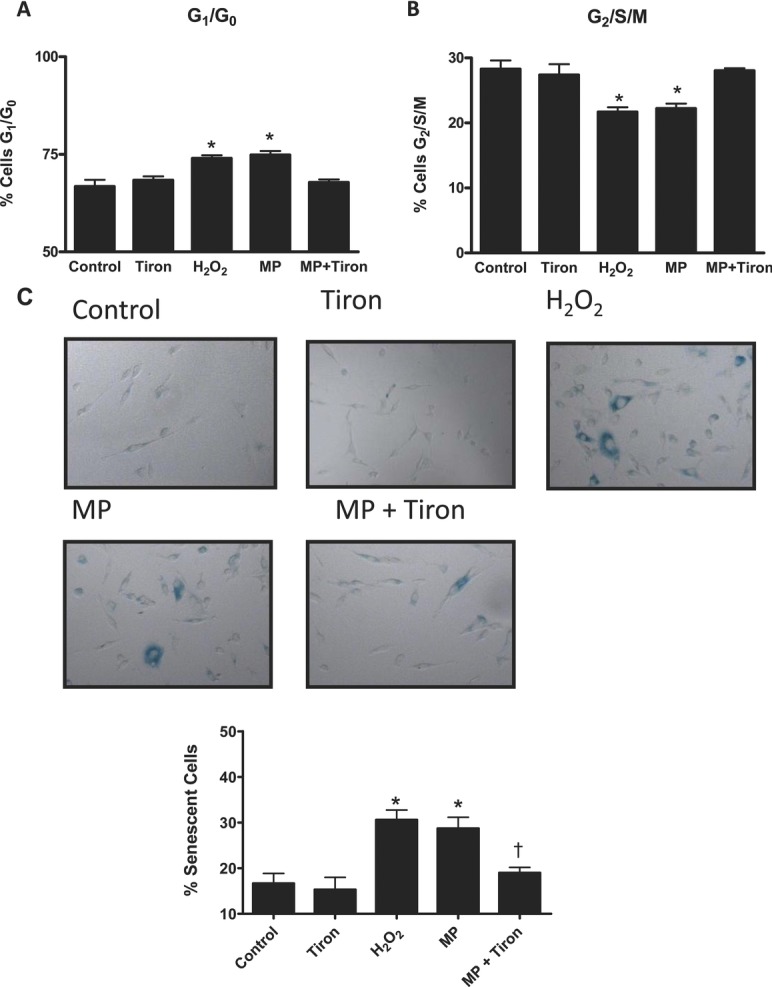
Cell cycle progression and SA-βgal activity in ECs treated with H_2_O_2_ (100 μmol/L), or MPs (10^5^/mL). A, Treatment with MPs or H_2_O_2_ for 8 hours increased the proportion of nonproliferative cells (G_1_/G_0_). B, Treatment with MPs or H_2_O_2_ for 8 hours significantly decreased the proportion of proliferating cells (G_2_/M). C, SA-βgal staining of ECs after 48 hours. Upper panels: Representative images show SA-βgal staining in ECs exposed to 4,5-dihydroxybenzene-1,3-disulfonate (tiron), H_2_O_2_ (positive control), and MPs in the absence and presence of tiron pretreatment. Lower panel is a bar graph of SA-βgal staining expressed as a percentage of the total EC population and presented as means±SEM of 4 to 5 experiments. Treatment with MPs or H_2_O_2_ increased the number of cells exhibiting SA-βgal activity. Treatment with the superoxide scavenger sodium tiron (10 μmol/L) alone had no effect on cell cycle progression (A, B) or SA-βgal activity (C), but cotreatment with MPs blocked MP-mediated reductions in proliferation and increases in SA-βgal activity. Data are expressed as mean±SEM; *P<0.05 vs control, *P<0.05 vs MP treatment, n=5.

**Figure 7.**
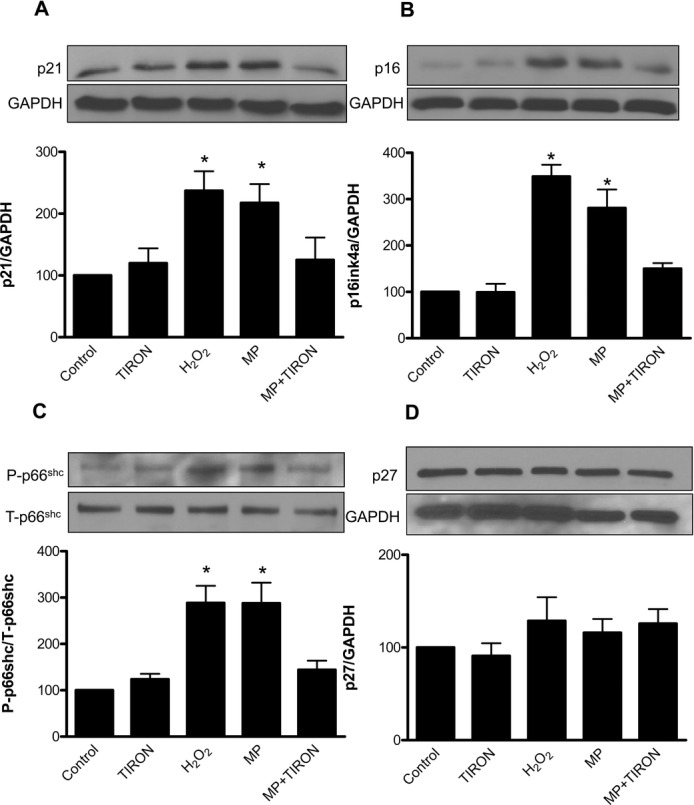
Effects of MPs on cell cycle and markers of cell senescence in ECs. Effect of MPs (10^5^/mL, 8 hours), H_2_O_2_ (100 μmol/L, 8 hours), and sodium 4,5-dihydroxybenzene-1,3-disulfonate (tiron, 10 μmol/L, 8 hours) on the expression of p21cip1 (p21), p16ink4a (p16), and p27kip1 (p27) and phosphorylation of p66^Shc^. Treatment with MPs or H_2_O_2_ increased expression of p21 (A) and p16 (B), increased phosphorylation of p66^Shc^ (C), but had no effect on p27 expression (D). Treatment with the superoxide scavenger sodium 4,5-dihydroxybenzene-1,3-disulfonate (tiron, 10 μmol/L) had no significant effect on SA-βgal staining, but cotreatment with MPs blocked MP-mediated effects on p21, p16, and p66^Shc^. Data are expressed as mean±SEM; *P<0.05 vs control, n=6 to 8.

To examine whether endothelial MPs contribute to premature EC senescence, mouse aortic ECs (p4 to p5) were treated with MPs obtained from low-passage ECs (10^5^ MPs/mL). As a positive control, ECs were treated with 100 μmol/L H_2_O_2_, which has been previously shown to induce premature senescence in ECs.^8,28^ Neither H_2_O_2_ nor MPs had any effect on apoptosis as determined by caspase-3 activity or Bax/Bcl-2 ratio (data not shown). However, treatment with MPs or H_2_O_2_ was associated with a shift from a proliferating to a nonproliferating phenotype as determined by propidium iodide DNA staining (Figure 6A and 6B). In addition, the expression of the cyclin-dependent kinase (Cdk) inhibitors p21cip and p16ink4a was significantly increased after treatment with H_2_O_2_ or MPs at 8 hours (Figure 7A and 7B). Similarly, phosphorylation of the longevity determinant adaptor protein p66^Shc^, which is both activated by ROS and a stimulus for ROS production, was increased after treatment with H_2_O_2_ or MPs (Figure 7C). Expression of p27kip1, a separate Cdk inhibitor, was not changed after treatment (Figure 7D). Finally, treatment with endothelial MPs was associated with induction of premature EC senescence, typified by an increase in the proportion of cells staining positive (bright blue) for β-galactosidase activity at pH 6 for 48 hours (*P*<0.05, Figure 6C).

### MPs Induce EC Senescence via a ROS-Dependent Pathway

As MPs are known to induce ROS production, and MP treatment was associated with phosphorylation of the ROS-sensitive protein p66^Shc^, implicated in senescence, we hypothesized that MP-mediated induction of EC senescence was dependent on ROS production. Using tiron, a O_2_^•−^ scavenger, we examined whether MP-induced premature EC senescence in p4 ECs is due to ROS production. Tiron treatment (8 hours) alone caused no changes in cell cycle progression but attenuated MP-mediated increases in nonproliferative G_0_/G_1_ populations (Figure 6A and 6B). Similarly, tiron blocked MP-induced increases in p21cip1 and p16ink4a expression and p66^Shc^ phosphorylation (Figure 7A through 7C). Finally, tiron treatment (48 hours) alone had no effect on SA-βgal activity but attenuated MP-induced increases in SA-βgal activity (Figure 6C).

### Mechanisms of MP-Mediated Increases in Oxidative Stress

**Figure 8.**
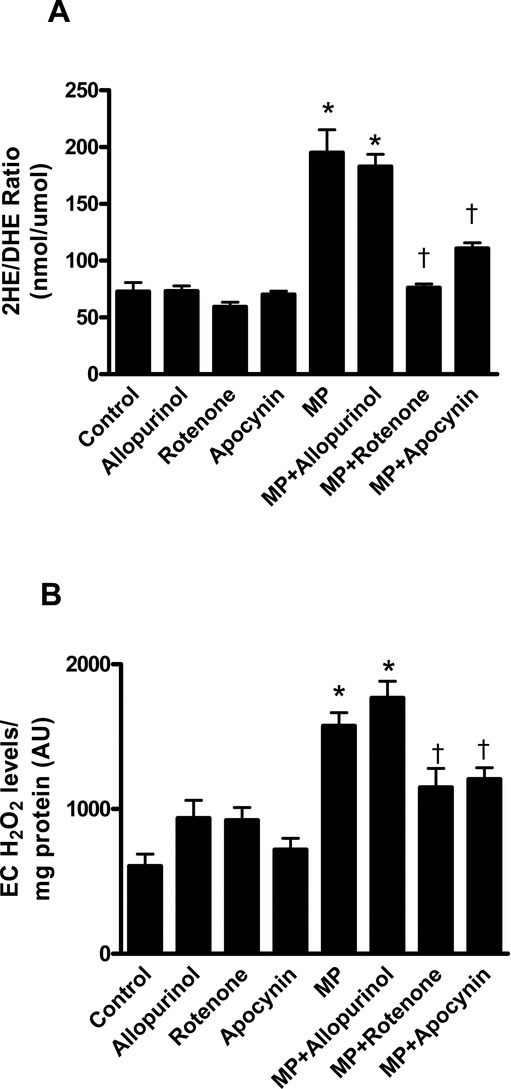
Role of prooxidant systems in MP-mediated oxidative stress. ECs were cultured and treated with endothelial MPs (10 ^5^/mL) for 4 hours in the presence and absence of apocynin (10 μM), rotenone (5 μM), or allopurinol (100 μM). Treatment with MPs significantly increased EC superoxide (O_2_^•−^, A) and hydrogen peroxide (H_2_O_2_, B) production as measured by DHE/HPLC (A) and Amplex Red (B), respectively. Cotreatment with apocynin and rotenone diminished MP-mediated increases in O_2_^•−^ and H_2_O_2_ production. Data are expressed as mean±SEM; *P<0.05 vs untreated control, *P<0.05 vs MP treatment, n=6.

Finally, to determine the mechanisms by which MPs promote oxidative stress in ECs, mouse aortic ECs (p4 to p5) were treated with MPs obtained from low-passage ECs (10^5^ MPs/mL) in the presence of inhibitors of NADPH oxidase (apocynin, 10 μmol/L), xanthine oxidase (allopurinol, 100 μmol/L), and mitochondrial respiration (rotenone, 5 μmol/L). O_2_^•−^(Figure 8A) and H_2_O_2_ (Figure 8B) formation was measured by DHE-HPLC and Amplex Red, respectively. Treatment with MPs was associated with a significant increase in EC ROS production. Cotreatment with allopurinol had no effect on MP-mediated increases in ROS production (Figure 8). Conversely, cotreatment with both apocynin and rotenone significantly reduced EC O_2_^•−^ and H_2_O_2_ production, suggesting that both NADPH oxidase and mitochondria, but not xanthine oxidase, play a role in MP-mediated increase in oxidative stress.

## Discussion

**Figure 9.**
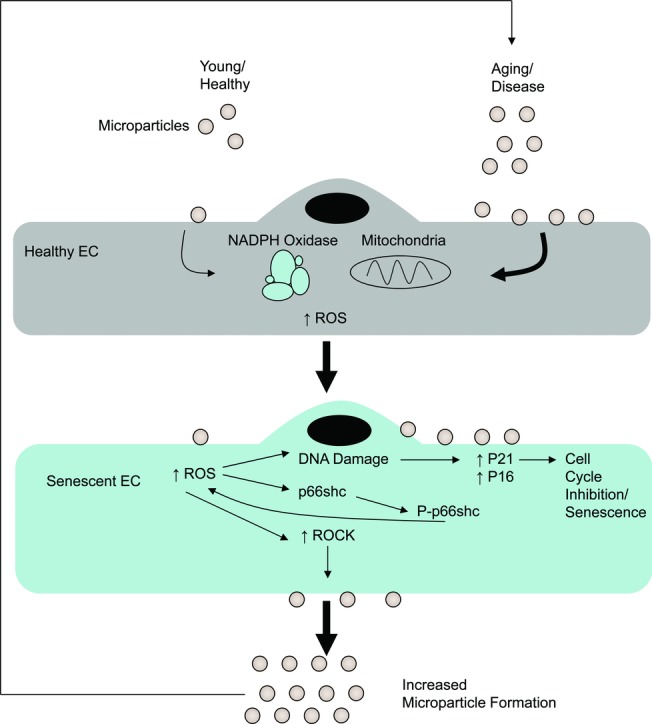
Putative mechanisms whereby MPs influence vascular cell aging and senescence. MP formation is increased in aging or under stress (disease) conditions, MPs, in turn, promote oxidative stress in ECs via NADPH oxidase and mitochondria. Increased oxidative stress ultimately results in EC senescence through DNA damage, leading to expression of cell cycle inhibitors p16ink4a (p16) and p21cip1 (p21) and increased activation of p66^Shc^, a determinant of cell longevity. The resultant EC senescence leads to increased MP formation via ROCK, which further promotes oxidative stress in a feed-forward fashion.

The present study examined the role of endothelial MPs in EC senescence. Major findings demonstrate that (1) long-term culture of mouse aortic ECs leads to a senescent phenotype associated with increased ROCK activity and formation of MPs, (2) MPs stimulate endothelial ROS production via NADPH oxidase and mitochondrial respiratory enzymes, and (3) MPs induce premature EC senescence in a ROS-dependent manner. Our results suggest that MPs contribute to the progression of vascular aging via a feed-forward mechanism wherein increased MP formation from senescent ECs promotes further EC senescence through increased ROS production. These processes are associated with upregulation of cell cycle inhibitor proteins (Figure 9).

MPs are increasingly recognized as cellular biomarkers of vascular injury/dysfunction in many pathologies (eg, hypertension, diabetes, end-stage renal disease).^14^ Despite this, little is known about the relationship between MPs and vascular senescence, and there are conflicting reports with regard to plasma levels of MPs during aging. On the one hand, P-selectin–expressing platelet MPs increase with older age in otherwise healthy individuals, ^29^ endothelial MPs are increased with age in diabetic rats, ^30^ and leukocyte-derived MPs are increased in thrombosed aged mice.^31^ Conversely, Forest et al reported that endothelial MPs decrease in individuals aged 75 years and older.^32^ Each of these studies involved in vivo measurement of plasma MP levels, where MP clearance may be a significant determinant of concentration. As aging is known to impair clearance of apoptotic cells, ^33^ we examined MP formation in vitro using a model of endothelial senescence through long-term culture as previously detailed. ^25,26^ Long-term culture of mouse aortic ECs led to phenotypic characteristics of aging/senescence, including decreased cell proliferation and increased SA-βgal staining. In addition to these phenotypic changes, MP release into media was significantly increased in high-passage ECs, suggesting that MP formation increases in senescent cells. This increase appears to be mediated through ROCK, as treatment with fasudil blocked MP formation in these cells, consistent with our previous work.^17^ Levels of the autophagy-associated protein LC3-II were decreased in high-passage ECs. This observation is consistent with age-related declines in autophagy, which have been reported elsewhere. ^34,35^ It has been speculated that MP formation represents an adaptation that allows healthy cells to rid themselves of deleterious/toxic components.^36^ On the basis of our observations, it is conceivable that the increase in MP formation in late-passage ECs represents an adaptive response to compensate for reduced autophagic degradation of undesirable cell components.

Consistent with previous reports, we observed that MPs derived from low-passage and high-passage ECs promote the production of ROS (O_2_^•−^ and H_2_O_2_) in cultured ECs.^16,17,18,37^ Despite the established pro-oxidative effects of MPs, the mechanisms responsible for ROS production after MP exposure have been elusive, and reports are contradictory. Lymphocytic MPs have been reported to promote EC ROS production via xanthine oxidase ^38^ or through NADPH oxidase.^39^ Monocyte MPs stimulate EC ROS production via NADPH oxidase, mitochondria, xanthine oxidase, cyclooxygenase, and uncoupled nitric oxide synthase.^40^ Recently, Terrisse et al implicated xanthine oxidase and possibly NADPH oxidase in endothelial MP-mediated EC ROS production.^18^ Our results suggest that these increases in EC ROS production are mediated via NADPH oxidase and mitochondria but not by xanthine oxidase. Reasons for the different results are unclear, but they may relate to differences in MP isolation/handling by various laboratories or to differences in the EC populations being treated.

Surprisingly, despite clear phenotypic differences between low- and high-passage ECs, MPs derived from both origins had a similar capacity for inducing ROS production in ECs. While others reported divergent biological activity of distinct MP populations,^41^ we found that endothelial MPs obtained from high-passage senescent ECs appear to promote EC oxidative stress to a similar extent to those obtained from low-passage ECs. In support of this, electron microscopy and Western blot analysis revealed that MPs derived from high-passage ECs were similar in composition to MPs derived from low-passage ECs. Thus, MP production appears to increase in senescent ECs while retaining similar biological activity, the product of which would be an increase in MP-mediated pro-oxidative effects during aging.

Under conditions of stress/damage, such as those that occur during oxidative stress or angiotensin II exposure, ECs can become prematurely senescent, which, in turn, may contribute to the early vascular aging seen in chronic disease.^42^ To determine whether endothelial MPs promote premature EC senescence, we exposed cultured ECs to MPs and examined effects on proliferation, the expression of cell cycle inhibitory proteins, and SA-βgal activity. H_2_O_2_, previously reported to rapidly induce a senescent phenotype in ECs, was used as a positive control for stress-induced premature EC senescence.^8,28^ Consistent with previous reports, H_2_O_2_ rapidly induced a reduction in EC proliferation as determined by propidium iodide cell cycle analysis and a significant increase in the number of ECs staining positive for SA-βgal activity. This stress-induced senescent phenotype was recapitulated in ECs treated with MPs. In addition, MP treatment promoted phosphorylation and activation of the mitochondrial adaptor protein p66^Shc^, a determinant of mitochondrial ROS production and longevity.^12,43,44^ On the basis of these observations, we hypothesized that MPs promote premature EC senescence through the stimulation of EC ROS production. To address this question, we treated ECs with MPs in the presence of the O_2_^•−^ scavenger tiron. Cotreatment with tiron blocked MP-induced reductions in proliferation and MP-induced increases in SA-βgal activity. Thus, MPs promote premature EC senescence through ROS-dependent processes.

Attenuation of cell cycle progression represents a central mechanism in the development of growth arrest and cell senescence. We therefore examined the effect of MP administration on the expression of the Cdk inhibitors p16(ink4a), p21(cip1), and p27kip1. p16ink4a inhibits Cdk 4, thereby reducing phosphorylation of retinoblastoma protein (Rb) and leading to a reduction in pro-proliferative transcription factor E2F-activity.^45^ p21cip1, a Cdk inhibitor activated by DNA damage and p53, is capable of binding and preventing the activation of cyclin E-Cdk2 and cyclin D-Cdk4, therein promoting growth arrest and senescence.^45^ p27kip1 functions very similarly to p21cip1, but its regulation is generally not due to endogenous DNA damage but rather through various cell surface signals, kinase activity, and microRNAs (miRNA).^46^ In the present study, H_2_O_2_ and MP-mediated stress-induced premature senescence was also associated with upregulation of cell cycle checkpoint pathways of p21cip1 and p16ink4a but not p27kip1. Cotreatment of MPs with tiron attenuated MP-mediated increases in p21cip1 and p16ink4a. Interestingly, p27kip1 expression was not upregulated in either H_2_O_2_- or MP-treated cells. While one might expect ROS-dependent upregulation of p27kip1, Deshpande et al have reported induction of p21cip1, but not p27kip1 after H_2_O_2_ administration in vascular smooth muscle cells, suggesting that ROS-dependent induction of cell cycle arrest in the vasculature may not involve changes in p27kip1 expression.^21^

Mechanisms whereby MP–EC interactions are transduced into intracellular changes and functional responses remain unclear. However, emerging evidence has identified integrin activation, phosphatidylserine-dependent internalization of MPs, interleukin-1 receptor activation, sonic hedgehog signaling, and transfer of miRNAs as possible mediators of MP-induced biological effects.^18,47–50^ In addition, we recently reported a surface interaction involving epidermal growth factor receptor activation, which contributes to pro-oxidative and proinflammatory effects of MPs.^17^ While the mechanisms of MP-induced oxidative stress and senescence were not explicitly explored, it is plausible that a similar MP–EC interaction via epidermal growth factor receptor contributes to the ROS-dependent pro-senescent effects seen here.

In summary, we report that endothelial MP formation is increased in senescent ECs and that endothelial MPs induce premature senescence in cultured mouse aortic ECs in a ROS-dependent manner. These novel findings demonstrate a link between endothelial MPs and EC senescence. This phenomenon sheds new light on the role of MPs in vascular pathobiology and suggests that MPs may themselves be key mediators of early vascular aging by promoting cellular senescence.
